# Transient portal vein thrombosis in liver cirrhosis

**DOI:** 10.1186/s12916-018-1069-8

**Published:** 2018-06-05

**Authors:** Xingshun Qi, Xiaozhong Guo, Eric M. Yoshida, Nahum Méndez-Sánchez, Valerio De Stefano, Frank Tacke, Andrea Mancuso, Yasuhiko Sugawara, Sien-Sing Yang, Rolf Teschke, Ankur Arora, Dominique-Charles Valla

**Affiliations:** 1 Liver Cirrhosis Study Group, Department of Gastroenterology, General Hospital of Shenyang Military Area, No. 83 Wenhua Road, Shenyang, 110840 Liaoning Province China; 2 Division of Gastroenterology, Vancouver General Hospital, Vancouver, BC Canada; 3 Liver Research Unit Medica Sur Clinic & Foundation, Mexico City, Mexico; 4 Institute of Hematology, Catholic University, Rome, Italy; 5 Department of Medicine III, RWTH Aachen University, Aachen, Germany; 6 Department of Internal Medicine, ARNAS Civico, Palermo, Italy; 7 Hepatology and Gastroenterology, Niguarda Ca’ Granda Hospital, Milan, Italy; 8 Department of Transplantation and Pediatric Surgery, Postgraduate School of Medical Science, Kumamoto University, Kumamoto, Japan; 9 Liver Unit, Cathay General Hospital and Fu-Jen Catholic University School of Medicine, Taipei, Taiwan; 10 Department of Internal Medicine II, Division of Gastroenterology and Hepatology, Klinikum Hanau, D-63450 Hanau, Germany; 11 Department of Radiology, Worthing Hospital, Western Sussex NHS Foundation Trust, West Sussex, UK; 12 Service d’hépatologie, Hôpital Beaujon, APHP, Clichy-la-Garenne, Paris, France; 13 Université Paris-Diderot and Inserm, Paris, France

**Keywords:** Portal vein thrombosis, Liver cirrhosis, Recanalization, Anticoagulation, Transient

## Abstract

In real-world clinical practice, the acceptance of anticoagulation therapy in the management of portal vein thrombosis (PVT) in patients with cirrhosis is limited by the fear of an increased bleeding risk. Additionally, accumulating evidence indicates that spontaneous recanalization of PVT may occur in the absence of antithrombotic treatment. Therefore, risk stratification based on outcomes in such patients is crucial for determining a therapeutic strategy. In this paper, we draw attention to the distinct clinical entity, “transient PVT” by introducing two cases with PVT that spontaneously recanalized in the absence of antithrombotic treatment. We reviewed the available data regarding the probability of and predictors for spontaneous recanalization of PVT. Available data suggest singling out transient thrombosis in the natural history of PVT in patients with cirrhosis because of its prognostic and management implications.

## Background

Anticoagulation therapy for portal vein thrombosis (PVT) may be considered in patients with cirrhosis based on the current practice guidelines and consensus statements [1, 2]. Indeed, two recently reported meta-analyses suggest that anticoagulation therapy improves the rate of portal vein recanalization and prevents thrombus progression in such patients [3, 4]. Still, the actual impact of recanalization on the clinical outcomes remains to be clarified. In real-world clinical practice, the acceptance of anticoagulation therapy for the management of PVT in patients with cirrhosis is limited by the fear of an increased risk of bleeding [5]. Recently, spontaneous recanalization of PVT has been reported by several investigators in patients with cirrhosis who did not receive any antithrombotic therapy [6–10]. Thus, some patients who develop spontaneous portal vein recanalization can avoid the exposure to anticoagulation and its related risk of bleeding.

There is also evidence that occlusive PVT is associated with a risk of bleeding from portal hypertension and death in patients with liver cirrhosis [11, 12]. However, available cross-sectional data do not allow clinicians to determine whether the link is causal. In comparison with occlusive thrombosis, the impact of partial PVT on the prognosis of cirrhosis may be marginal. If this proves to be true, the indication and timing for implementing anticoagulation therapy should be discussed accordingly.

Therefore, stratification along the degree of PVT in patients with cirrhosis could become important for guiding management, including a wait-and-see attitude, anticoagulation therapy, or transjugular intrahepatic portosystemic shunt (TIPS) [13].

### Key points

1. Risk stratification of portal vein thrombosis in liver cirrhosis should be widely acknowledged.
2. Spontaneous recanalization of portal vein thrombosis has been frequently observed in liver cirrhosis.
3. Transient portal vein thrombosis should be recognized as a distinct clinical entity in liver cirrhosis.
4. A watchful waiting should be considered in cirrhotic patients with recent portal vein thrombosis unaccompanied by clinical progression.
5. Further research should actively explore the predictors for transient portal vein thrombosis in liver cirrhosis.

## “Transient PVT” as a distinct entity

**Fig. 1:**
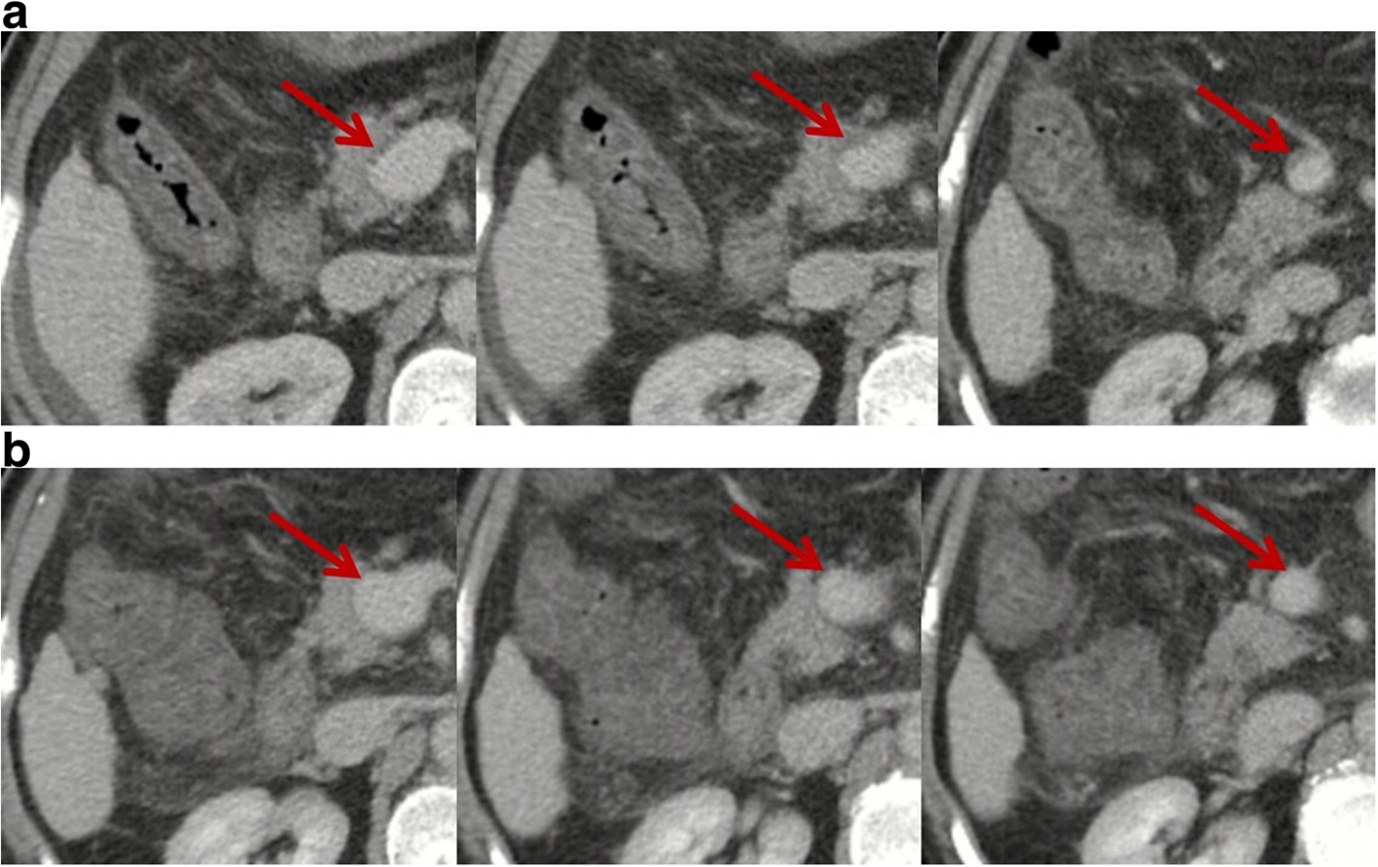
Contrast-enhanced computed tomography scans in a patient with transient PVT. Contrast-enhanced computed tomography performed in February 2017 demonstrated mild ascites, patent intrahepatic portal vein branches and splenic vein, mild thrombosis within main portal vein and superior mesenteric vein (SMV), and splenomegaly (panel **a**). *Red arrows* indicate mild thrombosis within the main portal vein and SMV. Notably, thrombus occupied less than 10% of the vessel lumen. In the absence of antithrombotic therapy, repeated contrast-enhanced computed tomography performed in April 2017 showed that intrahepatic portal vein branches, main portal vein, splenic vein, and SMV were patent (panel **b**). *Red arrows* indicate patent main portal vein and SMV

**Fig. 2:**
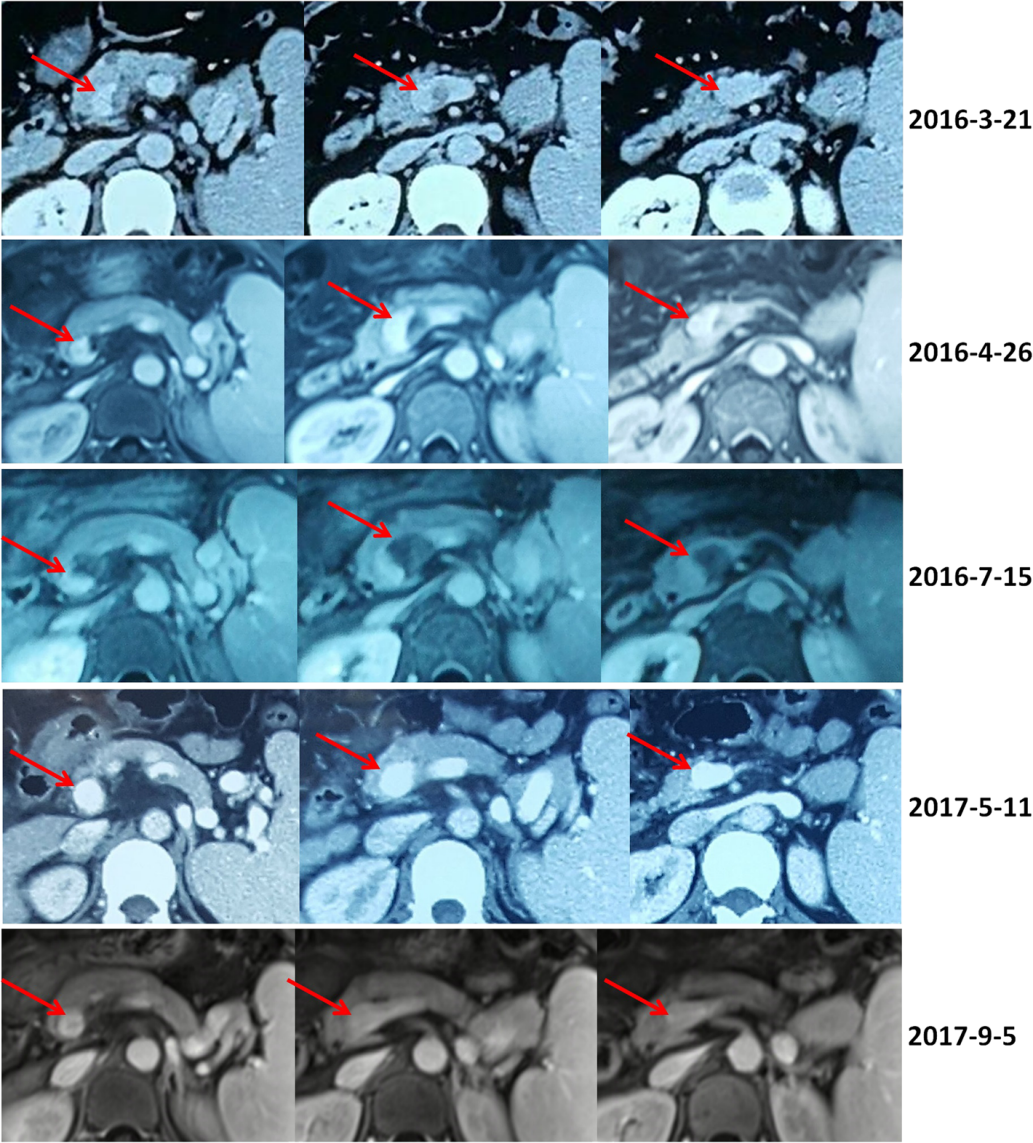
Contrast-enhanced computed tomography and magnetic resonance scans in a patient with transient PVT. Contrast-enhanced computed tomography and magnetic resonance scans performed in 2016 demonstrated a partial thrombosis within the confluence of portal vein and splenic vein (*red arrows*). Contrast-enhanced computed tomography and magnetic resonance scans performed in 2017 demonstrated that the confluence of portal vein and splenic vein was patent (*red arrows*)

The term “clinically significant PVT” has been proposed to identify the conditions in which the outcome of patients with cirrhosis is significantly compromised when PVT is present and, therefore, would benefit from antithrombotic treatment [14]. At the opposite end of the spectrum, some cases can develop spontaneous resolution of PVT in the absence of any antithrombotic treatment (Figs. 1 and 2). Thus, such “transient PVT” should be further singled out as a benign condition that might not warrant immediate treatment.

## Probability of transient PVT

Asymptomatic deep vein thrombosis has been well recognized. In about 1% of the healthy general population, a venous thrombus in the leg, most cases of which occur as an isolated calf vein thrombus, can be detected by ultrasonography and has no clinical consequences during follow-up [15]. Also, asymptomatic pelvic vein thrombosis following vaginal delivery does not appear to have clinical consequences [16]. In addition, asymptomatic venous thrombi following orthopedic surgery [17] and symptomatic distal venous thrombi [18, 19] may resolve spontaneously without anticoagulation, which suggests the probability of transient deep vein thrombosis. Such a phenomenon may also be observed in cirrhotic patients where the fibrinolytic pathway is deeply deranged (i.e., with increased tissue-type plasminogen activator and plasminogen activator inhibitor-1 levels and decreased plasminogen, alpha 2-antiplasmin, and thrombin-activatable fibrinolysis inhibitor levels) and a weak re-balance is established [20, 21].

Based on a previous systematic review of scientific publications regarding PVT [22], an updated search strategy of relevant items ([“portal vein thrombosis”] AND [“recanalization” OR “resolution”]) in PubMed and EMBASE databases, and the experiences of the current authors, we summarized the data from three case reports [23–25] and 14 cohort studies or randomized controlled trials [6–10, 26–34] allowing evaluation of “transient PVT” in Tables 1 and 2, respectively. Because we analyzed the natural history of PVT in cirrhosis, some papers exploring the probability of portal vein recanalization after TIPS [35, 36] or partial splenic embolization [37] were not considered in the current work.

**Table 1:** An overview of case reports regarding spontaneous recanalization of PVT in cirrhosis

First author, year (region)	Sex	Age (years)	Predisposing risk factors	Severity of thrombosis	Symptoms of thrombosis	Management	Outcomes	Interval from diagnosis to vessel patency
Borja, 2016 [25] (Singapore)	Female	60	Cryptogenic liver cirrhosis; invasive ductal carcinoma of left breast	Nonocclusive portal vein thrombus (< 50%)	Distended abdomen with ascites	Antibiotics; bedside ascitic fluid drainage; no antithrombotic treatment	Completely patent	11 months
Lai, 1997 [24] (Taiwan)	Male	67	Liver cirrhosis; esophageal transection and splenectomy	Nonocclusive portal vein thrombus within portal trunk and right portal vein	Ascites; icteric sclera	Conservative treatment with close observation; no antithrombotic treatment	Completely patent	2 months
Spahr, 1996 [23] (Canada)	Male	45	Alcoholic liver cirrhosis; accidental gallbladder puncture during transjugular liver biopsy	Thrombus within the right intrahepatic branch, left intrahepatic branch, main portal vein, and splenic vein (degree of thrombosis was unclear)	Slight epigastric discomfort	No invasive maneuvers, such as thrombolysis, were initiated. The patient was treated conservatively	Completely patent	7 weeks

**Table 2:** An overview of cohort studies or randomized controlled trials regarding outcomes of PVT in cirrhotic patients who did not receive any antithrombotic treatment

First author, year (region)	Type of study	Enrollment period	Target population	Follow-up duration	Degree of PVT	Location of PVT	No. Pts	Severity of liver cirrhosis	Proportion of HCC	No. spontaneous recanalization or improvement	No. thrombus progression
Hidaka, 2018 [26] (Japan)	Multi-center, randomized, double-blind, placebo-controlled, full-text	Oct 2014 – Mar 2016	LC with PVT	21 ± 3 days	> 50% of the cross-sectional lumen of the MPV, portal vein branches, SMV, or SV	MPV (*n* = 20) Portal vein branch (*n* = 11) SV (*n* = 0) SMV (*n* = 5)	36	Child-Pugh A/B/C: 6/24/6	6 (16.7%)	7 (19.4%)	7 (19.4%)
Chen, 2016 [27] (China)	Single-center, retrospective, controlled, full-text	Jan 2002 – Jun 2014	LC with PVT	Mean ± SD: 25.9 ± 23 months (for 36 patients)	MPV (0–25%/26–50%/51–75%/76–100%): 8/2/6/20 LPV (0–50%/51–100%): 12/24 RPV (0–50%/51–100%): 11/25 SV (splenectomy/0–50%/51–100%): 20/11/5 SMV (0–50%/51–100%): 17/19		16	Child-Pugh A/B/C: 8/21/2 Mean MELD score: 8.9 ± 3.01 (for 36 patients)	0 (0%)	4 (25%)	6 (37.5%)
Nery,^a^ 2015 [6] (France)	Multi-center, prospective, single-arm, full-text	Jun 2000 – Mar 2006	Child-Pugh A and B LC with de novo PVT	Mean ± SD: 65.2 ± 39.6 months	Non-occlusive at the first diagnosis of PVT (*n* = 101); occlusive at the first diagnosis of PVT (*n* = 17)	NA	101^b^	All patients had Child-Pugh A or B	0 (0%) (for baseline)	70 (70%) 89^c^ (89%)	14 (13.8%)
Chung, 2014 [28] (Korea)	Single-center, retrospective, matched, controlled, full-text	2003 – 2014	LC with non-malignant partial PVT	NA	Partial (*n* = 14)	MPV (n = 9) RPV (*n* = 7) LPV (*n* = 4) SMV (n = 2)	14	Child-Pugh A/B/C: 7/6/1	8 (57.1%)	5 (36%)	3 (21.4%)
Girleanu, 2014 [7] (Romania)	Single-center, prospective, single-arm, full-text	Jan 2011 – Oct 2013	LC with non-malignant partial PVT	Mean ± SD (range): 20.22 ± 8.61 (4–31) months	Partial	PV (*n* = 18) PV + SMV (*n* = 11) PV + SMV + SV (*n* = 1)	22	Child-Pugh A/B/C: 7/9/6 Mean MELD score: 12.73 ± 4.34	0 (0%)	5 (22.73%)	6 (27.27%)
Risso, 2014 [29] (Italy)	Single-center, retrospective, controlled, abstract	2005 – 2011	LC with pre-LT non-neoplastic PVT	NA	NA	NA	20	NA	NA	8 (40%)	NA
John, 2013 [8] (USA)	Single-center, prospective, single-arm, full-text	Jul 2004 – Jun 2009	LC awaiting LT and PVT	> 6 months in all patients	Partial (*n* = 38); occlusive (*n* = 32)	MPV, not SMV	70	Mean MELD score: 14.4 ± 5.0 (for 47 pts. with baseline PVT); 15.3 ± 6.7 (for 23 pts. with new PVT)	0 (0%)	22 (31.4%)	3 (4.3%)
Maruyama, 2013 [9] (Japan)	Single-center, retrospective, single-arm, full-text	Jan 1998 – May 2009	LC with de novo PVT	Mean ± SD (range): 65.2 ± 39.6 (0.87–136.4) months	Partial (*n* = 31); occlusive (*n* = 11)	Intrahepatic PV (*n* = 8) MPV (*n* = 15) SV (*n* = 6) MPV + intrahepatic PV (*n* = 9) MPV + SV (*n* = 2) MPV + SV + intrahepatic PV (*n* = 2)	42	Child-Pugh A/B/C: 14/22/6 Median MELD score: 10.6	0 (0%)	20 (47.6%)	3 (7.2%)
Caracciolo, 2013 [30] (Italy)	Single-center, retrospective, matched, controlled, abstract	NA	LC with partial PVT	6–12 months	Occlusion< 50% (*n* = 14)	NA	14	All patients had Child-Pugh A or B (no patient had Child-Pugh C)	0 (0%)	8 (57.6%)	NA
Luca, 2012 [10] (Italy)	Single-center, retrospective, single-arm, full-text	Jan 2004 – Dec 2009	LC with non-malignant, extrahepatic, partial PVT	Mean: 27 months; 3–6 months (*n* = 8); 6–12 months (*n* = 8); 12–24 months (*n* = 9); > 24 months (*n* = 19)	Occlusion: < 25% (*n* = 8) 25–50% (*n* = 11) 50–75% (*n* = 14) 75–90% (*n* = 9)	MPV (*n* = 15) MPV + SMV (*n* = 18) MPV + SMV + SV (*n* = 9)	42	Mean Child-Pugh score: 8.1 ± 1.9 Mean MELD score: 12.1 ± 2.9	0 (0%)	19 (45%)	20 (48%)
Senzolo, 2012 [31] (UK)	Single-center, prospective, controlled, full-text	Jan 2007 – Jan 2008	LC with PVT	Mean: 22.53 months	CTPV (*n* = 3) complete (*n* = 4) partial (*n* = 14)	Intrahepatic PV (*n* = 5) MPV (*n* = 21) SMV (*n* = 4) SV (*n* = 1)	21	Child-Pugh A/B/C: 5/9/7 Mean MELD score: 13.7 ± 3.6	0 (0%)	1 (5%)	15 (71.4%)
Garcovich, 2011 [32] (Italy)	Single-center, retrospective, matched, controlled, abstract	NA	LC with PVT	NA	Occlusion> 75% (*n* = 3)	NA	15	All patients had Child-Pugh A or B (no patient had Child-Pugh C)	0 (0%)	5 (33%)	NA
Francoz, 2005 [33] (France)	Single-center, retrospective, controlled, full-text	Jan 1996 – Dec 2001	LC awaiting LT and partial PVT	NA	Partial (*n* = 10)	MPV (*n* = 6) RPV (*n* = 4) LPV (*n* = 3)	10	Child-Pugh A/B/C: 2/6/2 Mean MELD score: 11.8 ± 6.2	2/38 (5.3%) (for 38 patients with SVT)	0 (0%)	6 (60%)
De Santis 2001 [34] (Italy)	Single-center, retrospective, single-arm, abstract	Jan 1996 – Dec 1999	LC with PVT and without HCC	NA	NA	NA	21	NA	0 (0%)	7 (33%)	NA

*Abbreviations: CT* computed tomography, *CTPV* cavernous transformation of the portal vein, *HCC* hepatocellular carcinoma, *LC* liver cirrhosis, *LPV* left portal vein, *LT* liver transplantation, *MELD* Model for End-Stage Liver Disease, *MPV* main portal vein, *NA* not available, *PV* portal vein, *PVT* portal vein thrombosis, *RPV* right portal vein, *SD* standard deviation, *SMV* superior mesenteric vein, *SV* splenic vein, *SVT* splanchnic vein thrombosis

^a^Six patients with PVT received anticoagulation

^b^Change of PVT in 101 patients with non-occlusive PVT, rather than those with occlusive PVT, was reported in the text

^c^Number of patients after adding 19 patients with PVT in whom thrombus spontaneously disappeared and then reappeared

**Fig. 3:**
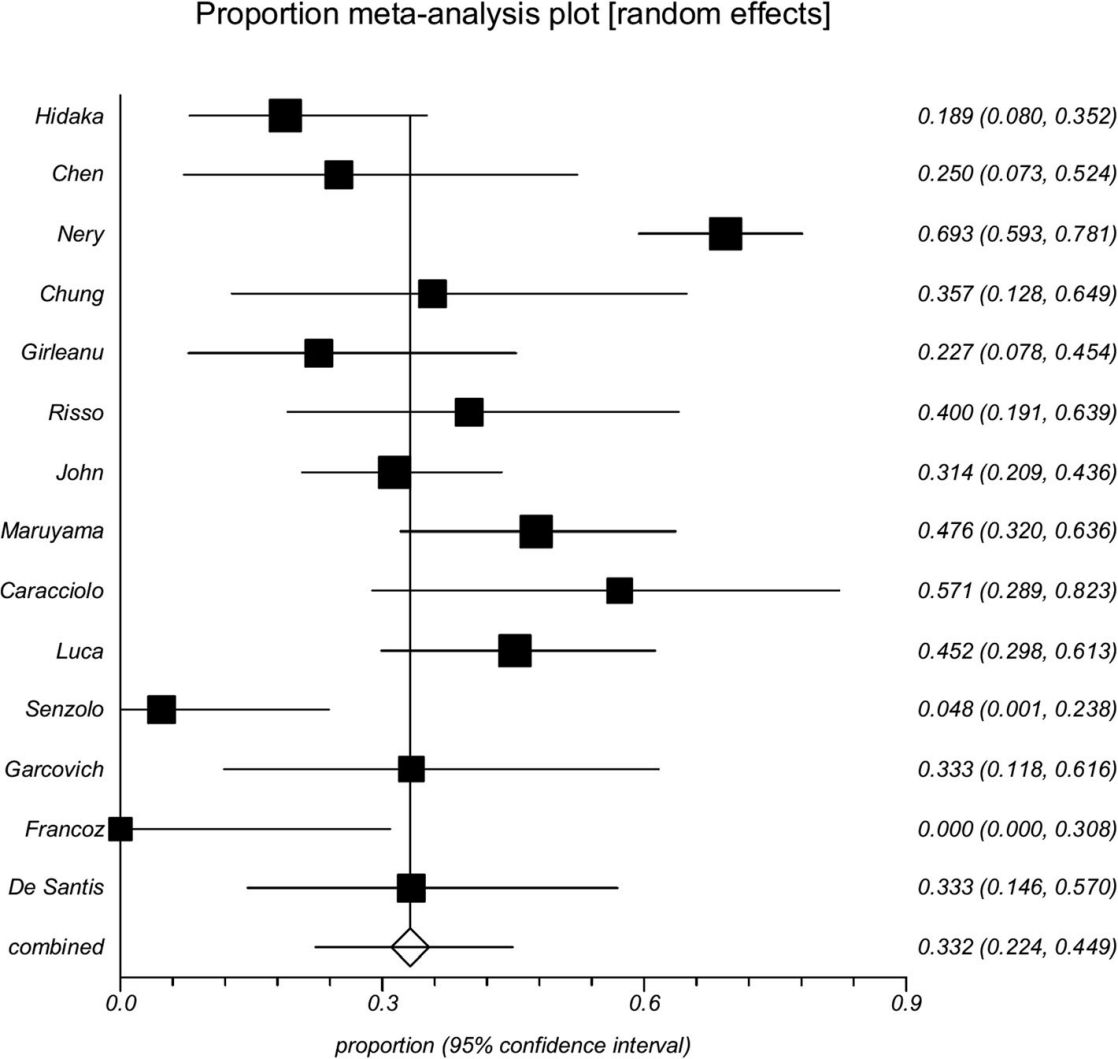
Forest plot of incidence of transient portal vein thrombosis in cirrhosis according to the data obtained from 14 studies

Among the 14 cohort studies or randomized controlled trials, 0–70% of PVT events were transient. The data were combined, and a proportion meta-analysis demonstrated that the pooled incidence of transient PVT was 39.8% (95% confidence interval 35.4–44.4%) (Fig. 3). There was a very remarkable heterogeneity among studies (Cochran Q = 82.09, *P* < 0.0001; *I*^2^ = 84.2%, 95% confidence interval 74.4–89.1%). The reasons why the incidence of spontaneous recanalization was very heterogeneous among studies merited analyses.

First, a majority of studies (64.3%, 9/14) were retrospective [9, 27–30, 32–34, 36]. Thus, the potential bias of patient selection should be acknowledged. For example, in the study by Chen et al. [27], 36 patients who did not receive anticoagulation were selected from 257 patients with cirrhosis and PVT. Furthermore, only 44.4% (16/36) of patients underwent the second computed tomography during follow-up to evaluate the portal vein recanalization. In the study by Chung et al. [28], 14 patients who did not receive anticoagulation were selected from 72 cirrhotic patients with PVT. In the study by Luca et al. [36], 42 eligible patients were selected from 178 cirrhotic patients with PVT. Maruyama et al. selected 150 patients with virus-related cirrhosis from 1964 patients with cirrhosis [9]. Notably, 341 patients were excluded due to insufficient laboratory data. Furthermore, 4 of the 9 retrospective studies were published in abstract form [29, 30, 32, 34], in which detailed information regarding patient characteristics, eligibility criteria, and extent/degree of PVT was unclear.

Second, two studies clearly included patients with hepatocellular carcinoma (HCC). In a study by Chung et al. [28], the proportion of HCC was up to 57%. The investigators stated that PVT should be non-malignant to be taken into account. In another study by Hidaka et al. [26], the proportion of HCC was 19.4%. The investigators stated that only patients with small HCC lesions were included.

Third, three studies included only patients with Child-Pugh class A and B [6, 30, 32] and reported a high incidence of spontaneous portal vein recanalization. A prospective cohort study by Nery et al. included mostly Child-Pugh class A patients and reported a high incidence of spontaneous portal vein recanalization in partial PVT (70/101, 70%) [6]. Although the incidence of spontaneous recanalization in occlusive PVT was not explicitly reported in the text, it could be estimated to 58.8% (10/17) based on the supplementary material. Another two studies by Caracciolo [30] and Garcovich [32] reported that the incidence of spontaneous portal vein recanalization was 57.6% (8/14) and 33% (5/15), respectively. As previously shown, the severity of liver dysfunction was negatively associated with the portal vein flow velocity [38], but positively associated with the risk of PVT [39]. It is imaginable that spontaneous portal vein recanalization may be easier in patients with relatively well-preserved liver function.

Fourth, two non-randomized controlled studies reported a very low probability of transient PVT in patients who did not receive any antithrombotic treatment. Francoz et al. conducted a retrospective comparative study in patients listed for liver transplantation and did not observe any events of spontaneous portal vein recanalization (0/10, 0%) [33]. We noted that patients on the waiting list for transplantation typically had more advanced, decompensated cirrhosis or HCC than those who were not listed for transplantation. Additionally, thrombus progression developed in a high proportion of patients (6/10, 60%). It should be noted that all patients who did not receive any antithrombotic treatment had partial PVT, rather than occlusive PVT. Information regarding follow-up duration and portal vein imaging plans was not provided. Similarly, another prospective comparative study by Senzolo et al. also observed a low incidence of spontaneous portal vein recanalization (1/21, 5%) and a high incidence of thrombus progression (15/21, 71.4%) [31]. We note that a majority of patients who did not receive any antithrombolytic treatment had partial PVT (67%). Because both comparative studies aimed to explore the efficacy of anticoagulation for PVT in liver cirrhosis, the potential selection bias of control group should not be ignored.

Therefore, the study design, patient characteristics, inclusion or exclusion of HCC, and severity of liver dysfunction and PVT should be carefully considered in every individual study when analyzing the probability of transient PVT.

## Impact of transient PVT on clinical outcomes

Several studies evaluated the prognostic impact of de novo PVT in liver cirrhosis [6, 8, 9]. Nearly all of them suggested no or marginal impact on the outcomes of liver cirrhosis. Only one retrospective study by Luca et al. analyzed the association of dynamic change of PVT with clinical outcomes [10]. The investigators divided patients into improved and stable/worsened PVT groups and found that spontaneous improvement of PVT did not provide any benefit in terms of the development of liver cirrhosis-related complications, liver transplantation, and survival. Multivariate analysis showed that the severity of cirrhosis as indicated by the Child-Pugh score at diagnosis was the only independent predictor of survival and hepatic decompensation. Therefore, current data suggested that the management of liver cirrhosis and its major complications should be maintained regardless of change of PVT.

## Predictors for transient PVT

The data regarding predictors for spontaneous recanalization or improvement of PVT are very scarce. Some features from scattered case reports (Table 1) are summarized [23–25]. First, two patients had transient risk factors for PVT, such as recent surgery (accidental gallbladder puncture during transjugular liver biopsy in the case reported by Spahr et al. [23] and splenectomy in the case reported by Lai et al. [24]). Unfortunately, the presence of transient risk factors provoking PVT is not specified in the cohort studies. Second, two patients had non-occlusive PVT [24, 25], and one patient had no information regarding the degree of PVT [23]. Thus, PVT may be mild or moderate in patients who developed spontaneous recanalization of PVT. Third, two patients developed spontaneous recanalization of PVT within 2 months after the diagnosis [23, 24] and one patient within one year [25]. PVT may spontaneously disappear during the short-term follow-up.

Furthermore, three cohort studies performed statistical analyses regarding predictors for spontaneous portal vein recanalization (Table 2). Their statistical results are also summarized. Luca et al. analyzed the associations of thrombus age (de novo vs. past PVT) and baseline clinical characteristics with regression of PVT [10]. No associated factors were found. Chen et al. also performed a univariate analysis to explore the baseline predictors for spontaneous recanalization of PVT [27]. Baseline predictors entered in the univariate analysis included age, sex, severity of liver and renal function (i.e., bilirubin, albumin, prothrombin time, creatinine, and Child-Pugh and Model for End-Stage Liver Disease (MELD) scores), esophageal varices, previous portal hypertension-related bleeding, ascites and decompensation, location of thrombosis (i.e., main portal vein, left portal vein, right portal vein, splenic vein, and SMV), and portal cavernoma. However, no significant predictors were identified. Maruyama et al. evaluated the predictive role of clinical and ultrasonic parameters in 42 patients with de novo PVT [9]. Univariate analysis demonstrated that the diameter and flow volume in the largest collateral vessel at the time of diagnosis of PVT were negatively associated with spontaneous improvement of PVT (improved vs. stable/worsened: 3.6 mm vs. 7.7 mm; 141.1 ml/min vs. 451.6 ml/min).

In theory, the milder the severity of PVT, the higher the likelihood of spontaneous portal vein recanalization. Maruyama et al. provided raw data regarding association of baseline degree (partial/non-occlusive vs. complete/occlusive) and location (intrahepatic portal vein branch vs. portal trunk vs. splenic vein vs. multiple vessels) of PVT with follow-up evolution of PVT [9]. Neither degree nor extension of PVT was significantly associated with change of PVT. The proportion of partial thrombosis was very similar between patients with improved and stable/worsened PVT (80% [16/20] vs. 81.8% [18/22], *P* = 0.881). The proportion of involvement of multiple vessels was slightly lower in patients with improved PVT than in those with stable/worsened PVT (25% [5/20] vs. 36.4% [8/22], *P* = 0.426). Notably, no SMV involvement was observed in the Maruyama study [9]. As recommended by the European Association for the Study of the Liver (EASL) Clinical Practice Guidelines [1], patients with SMV involvement may be a specific group of patients who need more aggressive antithrombotic treatment. Therefore, Maruyama’s findings should be cautiously interpreted. In addition, as mentioned above, two Italian studies by Caracciolo [30] and Garcovich [32] from the same affiliation provided data according to the degree of PVT. Although the incidence of spontaneous portal vein recanalization was higher in patients with a thrombus occupation of < 50% than in those with a thrombus occupation of > 75% (57.6% [8/14] vs. 33% [5/15]), no statistically significant difference was observed (*P* = 0.198).

All in all, except for the ultrasonic parameters identified by Maruyama et al. that require prospective external validation [9], no predictors for resolution of PVT have been identified. In the future, the candidates for watchful surveillance without anticoagulation therapy should be actively explored.

## Recurrence of PVT after spontaneous recanalization

Two cohort studies reported close follow-up surveillance data regarding thrombus recurrence after spontaneous recanalization of PVT. In the retrospective cohort study by Maruyama et al. [9], spontaneous portal vein recanalization was observed in 20 of 43 cirrhotic patients with PVT. Among the 20 patients, 9 (45%) developed a recurrence of PVT. The researchers did not identify any significant factors associated with the recurrence of PVT. In the prospective cohort study by Nery et al. [6], spontaneous portal vein recanalization after the diagnosis of PVT was observed in 89 of 101 patients with non-occlusive PVT. Among the 89 patients, 70 (78.7%) maintained the portal vein patency during the follow-up, but 19 (21.3%) developed a recurrence of PVT. Considering the possibility of thrombus recurrence, the patients should continue to monitor the portal vein patency after spontaneous portal vein recanalization. Of note, an international registry study suggested the lowest incidence of thrombotic recurrence in splanchnic vein thrombosis patients with transient risk factors [40].

## Conclusions

**Fig. 4:**
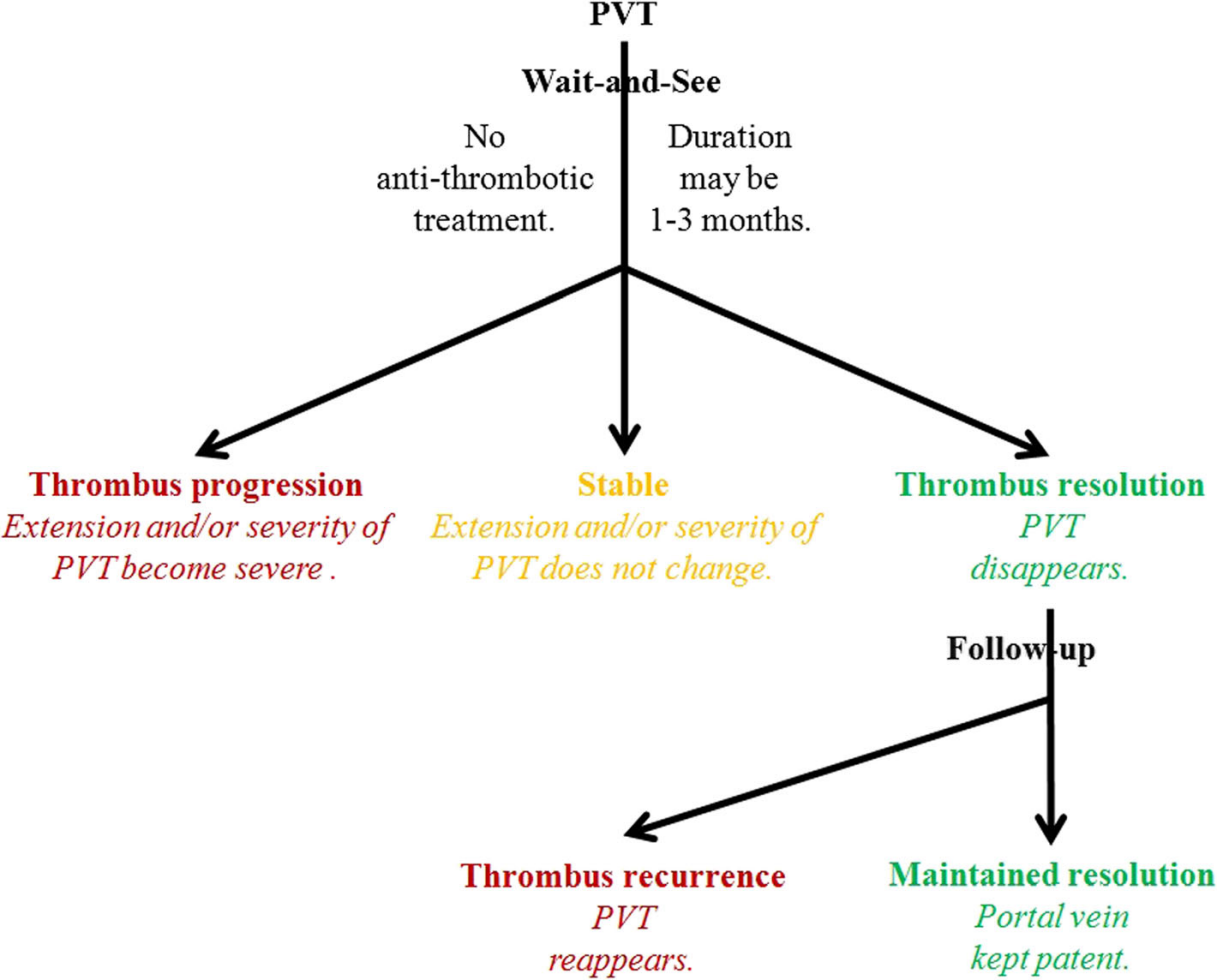
A preliminary diagram regarding the natural history of portal vein thrombosis in cirrhosis

Based on the current data, a diagram depicting the natural history of PVT in cirrhosis has been outlined to single out transient PVT (Fig. 4). Transient PVT should be defined if a thrombus within the portal vein spontaneously disappears during the short-term follow-up. According to the available prospective and longitudinal data [6], a definition for short-term follow-up may be less than 3 months. Preferably, the same (cross-sectional) imaging modality should be used to scan these patients for progression of PVT. We believe that the entity of transient PVT raises several issues requiring specific studies before a management scheme can be reasonably proposed: (1) the identification of accurate baseline predictors for spontaneous short-term recanalization (including the extent/degree of thrombosis); (2) the relationship of recanalization with clinical outcomes; and (3) the efficacy of anticoagulation therapy to prevent recurrent thrombosis and to impact the outcomes. If the clinical situation favors watchful waiting, patients with cirrhosis and recent PVT unaccompanied by clinical progression could be monitored monthly for 3 months for extension/stability/regression of thrombosis. Regarding candidates for liver transplantation, anticoagulation should be considered at the first documentation of an extension of the thrombus. Additionally, regardless of liver transplantation, anticoagulation might be prompted in patients with extension of thrombosis to SMV, with known thrombophilia, or with recurrent thrombosis in the absence of contraindications [1]. Further clinical research in this area is clearly required.

## Abbreviations

PVT: Portal vein thrombosis
SMV: Superior mesenteric vein
TIPS: Transjugular intrahepatic portosystemic shunt
